# Mechanical stress protects against osteoarthritis via regulation of the AMPK/NF‐κB signaling pathway

**DOI:** 10.1002/jcp.27592

**Published:** 2018-10-12

**Authors:** Yue Yang, Yang Wang, Yawei Kong, Xiaoning Zhang, He Zhang, Yi Gang, Lunhao Bai

**Affiliations:** ^1^ Department of Orthopedic Surgery Shengjing Hospital, China Medical University ShenYang Liaoning China; ^2^ Department of Ultrasound Shengjing Hospital, China Medical University ShenYang Liaoning China; ^3^ International Patient Center, Brigham and Women's Hospital, Harvard Medical School Boston Massachusetts

**Keywords:** AMP‐activated protein kinase (AMPK), chondrocytes, mechanical stress, nuclear factor (NF)‐κB, osteoarthritis (OA)

## Abstract

Mechanical stress plays a key role in regulating cartilage degradation in osteoarthritis (OA). The aim of this study was to evaluate the effects and mechanisms of mechanical stress on articular cartilage. A total of 80 male Sprague‐Dawley rats were randomly divided into eight groups (*n* = 10 for each group): control group (CG), OA group (OAG), and CG or OAG subjected to low‐, moderate‐, or high‐intensity treadmill exercise (CL, CM, CH, OAL, OAM, and OAH, respectively). Chondrocytes were obtained from the knee joints of rats; they were cultured on Bioflex 6‐well culture plates and subjected to different durations of cyclic tensile strain (CTS) with or without exposure to interleukin‐1β (IL‐1β). The results of the histological score, immunohistochemistry, enzyme‐linked immunosorbent assay, and western‐blot analyses indicated that there were no differences between CM and CG, but OAM showed therapeutic effects compared with OAG. However, CH and OAH experienced more cartilage damage than CG and OAG, respectively. CTS had no therapeutic effects on collagen II of normal chondrocytes, which is consistent with findings after treadmill exercise. However, CTS for 4 hr could alleviate the chondrocyte damage induced by IL‐1β by activating AMP‐activated protein kinase (AMPK) phosphorylation and suppressing nuclear translocation of nuclear factor (NF)‐κB p65. Our findings indicate that mechanical stress had no therapeutic effects on normal articular cartilage and chondrocytes; mechanical stress only caused damage with excessive stimulation. Still, moderate biomechanical stress could reduce sensitization to the inflammatory response of articular cartilage and chondrocytes through the AMPK/NF‐κB signaling pathway.

## 1 INTRODUCTION

Knee osteoarthritis (OA) is a highly prevalent, disabling joint disease. Its prevalence has doubled since the mid‐20th century, yet it is still poorly understood (Ondrésik et al., 2017; Wallace et al., 2017). OA is a degenerative joint disease characterized by articular cartilage degradation (Kalunian, 2016), which causes pain, stiffness, and even disability of joints (Garstang & Stitik, 2006; Wang et al., 2012).

Physical activity is one of the most widely applied nonpharmacological therapies for OA, but the duration and intensity of recommended exercise programs vary widely (Mcalindon et al., 2014). It is well accepted that different types of mechanical loading lead to different biological responses (Grad, Eglin, Alini, & Stoddart, 2011). Adequate exercise has been shown to benefit people with OA by relieving pain and increasing mobility (Barbour et al., 2014), but the pathology of OA is associated with excessive mechanical load. As mechanosensitive cells, chondrocytes synthesize the extracellular matrix and depend on intracellular signals generated in response to biomechanical stress (Harvey, Brosseau, & Herbert, 2014). Increasing evidence suggests that mechanical signaling plays a key role in regulating cartilage damage or repair. Despite active research in this area, it is still unclear how physical activity affects articular cartilage.

Several therapies aimed at ameliorating inflammatory response are currently being investigated. Molecular studies have revealed that specific biomechanical stimuli generate intracellular signals that are powerful inducers or suppressors of proinflammatory genes in chondrocytes (Knobloch, Madhavan, Nam, Agarwal, & Agarwal, 2008). Chondrocytes maintain a functional balance between degradation and repair by producing various enzymes, cytokines, and matrix‐associated proteins. A loss of this functional balance is associated with changes in the phenotypic characteristics of chondrocytes. Phenotypically, chondrocytes are characterized by their ability to synthesize collagen to withstand changes in their mechanical environment. Thus, the mechanisms by which chondrocytes convert biomechanical signals into intracellular biochemical events need further investigation.

Cyclic tensile strain (CTS) can be applied to cultured chondrocytes in a wide range of strain magnitudes, frequencies, and durations (Agarwal et al., 2004; Huang, Ballou, & Hasty, 2007; Kawakita et al., 2012; Long, Gassner, & Agarwal, 2001; Perera et al., 2010; Xu et al., 2011). The experimental setup is validated, controllable, and allows for the study of cell responses (Colombo, Cahill, & Lally, 2008). CTS also provides new insights into loading and cartilage adaptation. These mechanical signals acting on chondrocytes are critical regulators of tissue adaptation, structure, and function (Ramage, Nuki, & Salter, 2009). It is still unclear how intracellular signals generated by CTS of different durations with or without inflammatory stimulation produce these changes.

AMP‐activated protein kinase (AMPK) acts as an intracellular sensor that modulates the energy balance within chondrocytes. AMPK is exquisitely sensitive to the adenosine monophosphate (AMP)/adenosine triphosphate (ATP) ratio and intracellular calcium (Ca^2+^) levels. The role of AMPK is not only to regulate protein synthesis related to inflammation but also to modulate mitochondrial biogenesis (Gwinn et al., 2008). Inhibition of AMPK activation significantly impaired mitochondrial function and increased the generation of reactive oxygen species (ROS; Li, Wu, & Tian, 2018; X. Chen et al., 2018; Zhao & Yu, 2018). Further, AMPK responds to energy stress by regulating cell growth and biosynthetic processes, mainly through its inhibition of the nuclear factor (NF)‐κB signaling pathway. NF‐κB p65 is thought to be a link between tensile loading and the responses of chondrocytes to proinflammatory cytokines (Yang, Wang, Kong, Zhang, & Bai, 2017). Activation of NF‐κB p65 is a key event in matrix metalloproteinase (MMP) gene expression (Aupperle et al., 2001).

In this study, we evaluated the potential effects of different durations of treadmill exercise on cartilage with or without monoiodoacetate (MIA) injection. Furthermore, to identify potential contributions of mechanical stress at the cellular level, specifically the AMPK/NF‐κB signaling pathway, chondrocytes were subjected to CTS (0.5 Hz, 10%) of different durations with or without interleukin‐1β (IL‐1β).

## 2 MATERIALS AND METHODS

### 2.1 Experimental animals

A total of 80 male Sprague‐Dawley (SD) rats (230 ± 10 g; specific‐pathogen‐free) were obtained from HFK Bioscience Co. Ltd. (Beijing, China). This study was carried out in accordance with the recommendations of the Ethics Committee of Shengjing Hospital, China Medical University. The protocol was approved by this committee. Rats were kept in individual plastic cages on sawdust bedding; the environment included a 12 hr:12 hr light: dark cycle with the lights on from 6:00 a.m. to 6:00 p.m., a controlled temperature of 22 ± 2°C, and 70% humidity. The rats had free access to a planned diet. Body weight was recorded at regular intervals. They were adapted to laboratory conditions for 1 week before the experimental procedures. All rats were habituated to ZH‐PT treadmill exercise (Zhongshidichuang Science & Technology Development Co. Ltd., Beijing, China) for 1 week at a speed of 10 m/min for 10 min/day to reduce stress. All rats successfully adapted to the treadmill exercise.

### 2.2 OA model and treadmill running protocols

**Figure 1:**
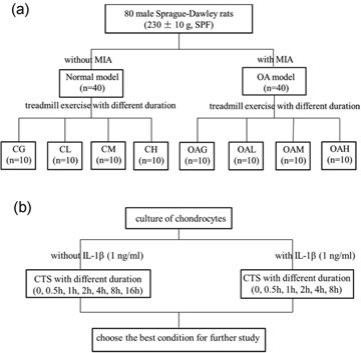
The design of the treatment schedule. Experimental groups: CG; CL, CM, and CH, control group subjected to different durations of treadmill exercise; OAG; and OAL, OAM, and OAH, OA subjected to different durations of treadmill exercise. CG: control group; CTS: cyclic tensile strain; IL‐1β: interleukin‐1β; MIA: monoiodoacetate; OA: osteoarthritis; OAG: OA group; SPF: specific‐pathogen‐free

After the adaptive treadmill exercise, the SD rats were numbered from 1 to 80 and randomly grouped by an Excel function into eight groups (*n* = 10 for each group): control group (CG); CG subjected to low‐, moderate‐, or high‐intensity treadmill exercise (CL, CM, and CH, respectively); knee OA model group (OAG); and OAG subjected to low‐, moderate‐, or high‐intensity treadmill exercise (OAL, OAM, and OAH, respectively).All rats were anesthetized with 1.5% pentobarbital sodium (30 mg/kg, intraperitoneal injection). Knee joint inflammation was induced by intra‐articular injection of MIA (1 mg per cavity in 50 μl sterile saline) by microsyringe through the infrapatellar ligament and into the bilateral knee joint cavity. The rats of CG, CL, CM, and CH received an intra‐articular injection of 50 μl sterile saline. CG and OAG rats were kept sedentary, but rats in the other groups began their exercise programs 24 hr after injection. The rats of CL and OAL exercised 30 min once daily, CM and OAM exercised 60 min once daily, and CH and OAH exercised 90 min once daily (19.3 m/min with 5° of inclination and 5 days/week for 4 weeks for all treadmill exercise) with appropriate photostimulation, acoustic stimulation, and electric stimulation (Figure 1).

### 2.3 Sampling and tissue preparation

After 4 weeks of treadmill exercise, all rats were anesthetized. Blood samples were obtained immediately after the animals were anesthetized, and the samples were centrifuged at 3,000 g for 10 min to obtain serum. Intra‐articular lavage fluid (IALF) was obtained from the synovial cavity of the right knee of each rat by injection and recovery of 200 μl of phosphate‐buffered saline (PBS) three times. All rats were then killed by cervical dislocation. The left knee joints of all rats were dissected and fixed in 4% paraformaldehyde solution. Articular cartilage was removed from the weight‐bearing area of the condyles of the right femur and tibia using a scalpel. All tissues were stored at −80°C until further analysis.

### 2.4 Histology

Left knee joint tissue samples were stored in 4% paraformaldehyde for 7 days. Then, they were washed in water for 5 hr and transferred to 20% EDTA solution (Jianglai Reagent Co., Ltd, Shanghai, China) to decalcify for 21 days; the solution was changed every 3 days. Decalcified samples were dehydrated in an ethanol series and embedded in paraffin. Serial 5‐μm sagittal sections were cut from the tibiofemoral joints for histological examination. The sections were stained with hematoxylin and eosin, as well as toluidine blue, to observe the cartilage. Next, the sections were visualized with ScanScope (APERIO CS2, Leica Biosystems Inc., Buffalo Grove, IL). Injuries to the articular cartilage in the femur and tibia were assessed by the Modified Mankin score (scale of 0–14 points; Pritzker et al., 2006) and the Osteoarthritis Research Society International (OARSI) score (scale of 0–24 points; Gerwin, Bendele, Glasson, & Carlson, 2010). Because both the tibial and femoral cartilages were evaluated, the maximum Mankin score was 28 and the maximum OARSI score was 48. Two experienced observers (Yue Yang and Xiaoning Zhang) performed the scoring in a blinded manner.

### 2.5 Immunohistochemistry

In addition to histomorphological evaluation, serial sections were stained for assessment of collagen II and MMP‐13 contents. After deparaffinization and rehydration of the tissue sections, endogenous peroxidase activity was blocked by 3% H_2_O_2_ for 20 min. The proteins were immunostained using a 2‐step method according to the kit manufacturer's instructions. The sections were incubated with rabbit polyclonal anti‐collagen II antibody (ab34712, 1:100; Abcam, Cambridge, MA), and rabbit polyclonal anti‐MMP‐13 antibody (ab39012, 1:50; Abcam) overnight at 4°C. The slides were washed three times in PBS followed by incubation for 20 min at 37°C with an anti‐mouse/rabbit immunoglobulin G (IgG) detection system (PV‐9000; Zhongshan Goldenbridge Biotechnology Co., China) and visualized with diaminobenzidine. Nuclei were counterstained with hematoxylin for 5 min. The optical densities of the stained slides were measured using image analysis software (Nikon H600L Microscope and image analysis system, Japan). Collagen II was expressed by relative intensity. MMP‐13 was expressed by the percentage of positive cells.

### 2.6 Enzyme‐linked immunoassay of tumor necrosis factor‐α and IL‐1β

Tumor necrosis factor (TNF)‐α and IL‐1β levels in knee IALF and in serum were determined using enzyme‐linked immunosorbent assay (ELISA) kits (Tongwei Co. Ltd., Shanghai, China) according to the manufacturer's instructions. The content of protein in IALF was measured to ensure that the ratio of dilution was equal.

### 2.7 Western blot analysis

Cartilage was washed twice in ice‐cold PBS. Proteins in the cytoplasm and nucleus were isolated by using the Cytoplasmic and Nuclear Protein Extraction Kit (Wuhan Boster Biotechnology Ltd., Wuhan, China) according to the manufacturer's instructions. The protein concentrations in the cytoplasm and nucleus were measured with a bicinchoninic acid assay kit (Beyotime Institute of Biotechnology, China). Equal amounts of protein (40 μg) were separated by sodium dodecyl sulfate–polyacrylamide gel electrophoresis and transferred to polyvinylidene difluoride membranes. After blocking with 1% bovine serum albumin (BSA) in tris‐buffered saline (TBS) with 0.1% Tween‐20 (TBST) at room temperature for 2 hr, the blots were incubated overnight at 4°C with primary antibodies: rabbit polyclonal anti‐ collagen II antibody (ab34712, 1:5,000; Abcam), molecular weight 142 kDa; rabbit polyclonal anti‐NF‐κB p65 antibody (AB21014, 1:500; Absci), molecular weight 65 kDa; rabbit polyclonal anti‐AMPK alpha 1 (phospho S487) antibody (ab131357, 1:500; Abcam), molecular weight 64 kDa; rabbit monoclonal anti‐AMPK alpha 1 antibody (ab32047, 1:3,000; Abcam), molecular weight 63 kDa; rabbit polyclonal anti‐MMP‐13 antibody (ab39012, 1:3,000; Abcam), molecular weight 54 kDa; and mouse monoclonal anti‐β‐actin (60008‐1‐lg, 1:5,000; Proteintech Group), molecular weight 42 kDa; rabbit monoclonal anti‐IκB‐α antibody (ab32518, 1:1,0000; Abcam), molecular weight 36 kDa; rabbit polyclonal anti‐histone H2A.X (AB41012, 1:1,000, Absci), molecular weight 19 kDa. After washing three times with TBST, the membranes were incubated with IgG‐horseradish peroxidase‐conjugated secondary antibodies (1:10,000; Canlife) at room temperature for 2 hr. After washing with TBST buffer, immunoreactivity was detected with enhanced chemiluminescence and quantified using Quantity ONE (Bio‐Rad, Hercules, CA) software. β‐actin or histone H2A.X was used as the internal control.

### 2.8 Isolation and culture of chondrocytes

Chondrocytes were obtained from the articular cartilage of knee joints of male SD rats (150 ± 10 g; specific‐pathogen‐free). Tissue was collected in sterile PBS. Articular cartilage pieces were incubated by sequential digestion with pronase (2 mg/ml) and collagenase D (1 mg/ml; Roche, Basel, Switzerland). Cells were cultured in 25‐cm^2^ cell‐culture flasks in Dulbecco's modified Eagle medium (Gibco BRL, Grand Island, NY) with 10% fetal bovine serum (Gibco BRL) and antibiotics (100 U/ml penicillin and 100 μg/ml streptomycin) in a humid atmosphere of 5% CO_2_ in air at 37°C. Upon reaching confluence, cells were detached with 0.25% trypsin and split in a 1:3 ratio. The cells were identified by immunohistochemical staining with anti‐collagen II antibody (ab34712, 1:100; Abcam; Figure 5a). For all experiments, the fourth through sixth passages were used. Using light microscopy, more than 95% of cells were judged to be chondrocytes.

### 2.9 Exposure of chondrocytes to CTS

Chondrocytes were grown on collagen I‐coated Bioflex 6‐well culture plates (Flexcell International, Hillsborough, NC) to 80%‐90% confluence. CTS experiments were performed using the FX‐5000 Flexcell system (Flexcell International, McKeesport, PA). To provide uniform radial and circumferential strain on the membranes, the plates were placed on a loading station (located in an incubator with 5% CO_2_) such that when a vacuum was applied to the loading station, the membrane deformed across the post face, creating uniform biaxial strain. Chondrocytes were subjected to CTS (10%, 0.5 Hz) for different durations (0, 0.5, 1, 2, 4, 8, and 16 hr) with or without IL‐1β for 24 hr. The stimulations of CTS and IL‐1β on chondrocytes began at the same time. We choose the best condition for further study. Compound C (ab120843; Abcam), a selective and reversible AMPK inhibitor, was used for pretreatment for 1 hr before the stimulation with IL‐1β and CTS (Dai et al., 2017).

### 2.10 Measurement of AMP, ADP, and ATP contents

Normal chondrocytes and chondrocytes subjected to CTS (10%, 0.5 Hz, 4 hr) were collected. The simultaneous determination of the contents of ATP, adenosine diphosphate (ADP), and AMP in the samples was accomplished by high‐performance liquid chromatography (HPLC; Bergamin et al., 2015). Briefly, cells were detached with 0.25% trypsin. The cells (3 wells of 6‐well culture plates) were washed with ice‐cold PBS three times and centrifuged to form a single pellet. The pellet was sonicated in 200 µl ice‐cold PBS and centrifuged. A supernatant (5 µl) was used for protein concentration determination. A supernatant150 µl) was also deproteinized by adding 150 µl ice‐cold methanol, vortexing thoroughly, and being placed on ice for 30 min. Next, the samples were centrifuged at 12,000*g* for 30 min at 4°C. The supernatant was passed through a 0.2‐µm filter. A supernatant (20 µl) was applied to a reversed‐phase C18 column (Kromasil C18, 5 µm, 4.6 × 250 mm; Phenomenex, Torrance, CA) on a Shimadzu LC‐10vp HPLC system (Shimadzu, Japan). The elution was carried out with 60 mM KH_2_PO_4_ plus 5% methanol at a rate of 1 ml/min. The amounts of AMP, ADP, and ATP were measured by absorption at 254 nm. Compound peaks from the samples were identified by the retention times and quantified by comparison of the peak areas of the samples with those of authentic standards.

### 2.11 Determinations of intracellular Ca^**2+**^ and ROS

Chondrocytes were seeded and cultured in 6‐well plates (1.5 × 10^6^ cells/well). Chondrocytes were exposed to IL‐1β (1 ng/ml) for 24 hr with or without different durations of CTS (10%, 0.5 Hz, 4 hr). The concentration of intracellular Ca^2+^ was detected by Fluo‐4AM (Dojindo, Kumamoto, Japan). Cells were then incubated with 2 μmol/L Fluo‐4AM in Hank's balanced salt solution for 30 min at 37°C in darkness.

The production of ROS was measured by 2′,7′‐dichlorodihydrofluorescein diacetate (DCFH‐DA; S0033; Beyotime), which is directly oxidized by ROS such as superoxide ion, hydrogen peroxide, and hydroxyl. For the DCFH‐DA assay, chondrocytes were incubated with 10 μM DCFH‐DA for 45 min at 37°C in darkness and then washed in PBS three times.

Fluorescence images were obtained using an OLYMPUS IX71 inverted microscope and analyzed with MetaFluor software 7.8 (Molecular Devices, Sunnyvale, CA). The ratio of fluorescence intensity (F/F0) was used to compare intracellular Ca^2+^ and ROS concentrations under different treatments (F: average fluorescence intensity under different treatments; F0: CG fluorescence intensity without any intervention; Sun, Yang, Ran, & Yang, 2016).

### 2.12 Immunofluorescence analysis of chondrocytes

After washing with PBS and being fixed with 4% paraformaldehyde for 20 min at room temperature, the cells were permeabilized with 0.5% Triton X‐100 for 30 min and incubated in nonspecific binding blocking solution (5% BSA) for 30 min at room temperature. Rabbit polyclonal anti‐NF‐κB p65 antibody (AB21014, 1:50; Absci) was added to the cells overnight at 4°C followed by staining with Alexa Fluor 488 conjugated anti‐rabbit antibody for 60 min at room temperature in darkness. The cytoskeleton was stained with phalloidine for 60 min at 37°C. Nuclei were counterstained with 4,6‐diamidino‐2‐phenylindole for 2 min. After washing, the cells adhered to Bioflex membranes were mounted in PBS with 20% glycerol. The chondrocytes were visualized with a confocal microscope (Olympus, Tokyo, Japan).

### 2.13 Statistical analysis

Data were analyzed using SPSS statistical software version 16 (SSPS, Inc., Chicago, IL). Results are expressed as means with 95% confidence intervals. Shapiro–Wilk's and Levene's tests were applied to evaluate the normality and homogeneity of the results, respectively. For variables that exhibited a normal distribution, independent samples *t* test and one‐way analysis of variance were used for the statistical analysis of significance. *p*‐values less than 0.05 were considered significant.

## 3 RESULTS

### 3.1 Histological observations and immunohistochemical analysis

**Figure 2:**
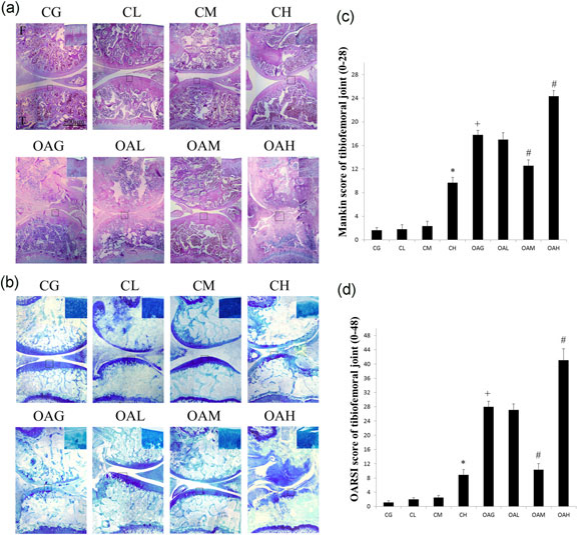
Histological evaluation of tibiofemoral joints. Histological features of representative tibiofemoral joints sectioned in the sagittal plane stained with HE (a) and toluidine blue (b). Mankin and OARSI histological scores are shown for each image. F: femur, T: tibia. (c) Mankin score of tibiofemoral joints. Differences between CG and CH (*p < 0.001), CG and OAG (^+^ *p* < 0.001), and OAG versus OAM and OAH (^#^ *p* < 0.001) were significant. (d) OARSI histological scores for cartilage of tibiofemoral joints. Differences between CG and CH (*p < 0.001), CG and OAG (^+^ *p* < 0.001), and OAG versus. OAM and OAH (^#^ *p* < 0.001) were significant. Results according to one‐way analysis of variance, presented as means with 95% confidence intervals; *n* = 10 rats in each group. Experimental groups: CG; CL, CM, and CH, control group subjected to different durations of treadmill exercise; OAG; and OAL, OAM, and OAH, OA subjected to different durations of treadmill exercise. CG: control group; OAG: OA group; OARSI: Osteoarthritis Research Society International [Color figure can be viewed at wileyonlinelibrary.com]

**Figure 3:**
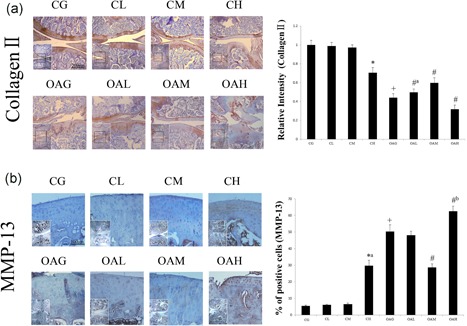
Immunohistochemical staining in each group. The micrographs show the relative intensities of immunohistochemical staining of collagen II (a) and the percentages of positively stained cells of MMP‐13 (b) in the articular cartilage of each experimental group. Differences between CG and CH (*p < 0.001), CG and OAG (^+^ *p* < 0.001), and OAG versus OAL, OAM, and OAH (^#^ *p* < 0.001, ^#a^ *p* = 0.019, ^#b^ *p* = 0.006) were significant. Results according to one‐way analysis of variance, presented as means with 95% confidence intervals; *n* = 5 rats in each group. Experimental groups: CG; CL, CM, and CH, control group subjected to different durations of treadmill exercise; OAG; and OAL, OAM, and OAH, OA subjected to different durations of treadmill exercise. CG: control group; OAG: OA group; MMP‐13: matrix metalloproteinase‐13 [Color figure can be viewed at wileyonlinelibrary.com]

Histological assessment (Mankin and OARSI score) and immunohistochemical staining (collagen II and MMP‐13) revealed that there were no differences among CL, CM, and CG, but OAM achieved therapeutic effects compared with OAG. CH and OAH showed evidence of potential cartilage damage compared with CG and OAG, respectively (Figures 2 and 3).

### 3.2 ELISA of TNF‐α and IL‐1β

**Figure 4:**
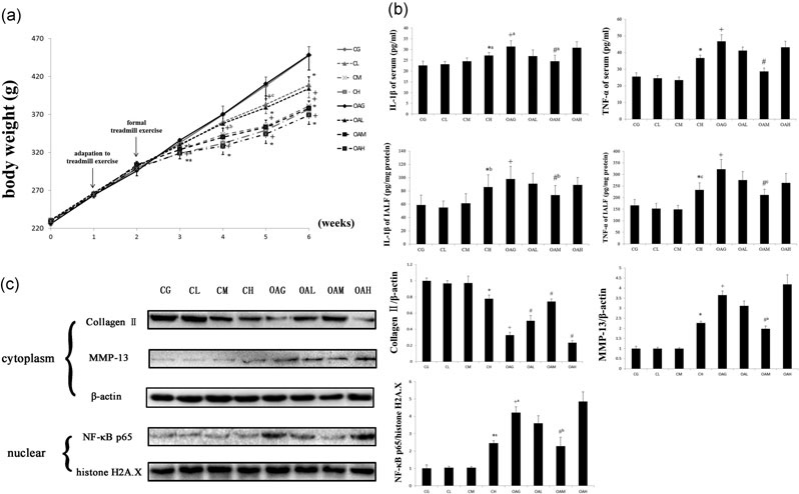
Comparisons of body weight, enzyme‐linked immunosorbent assay, and western blot analyses. (a) The results of body weight comparisons. The differences between CG versus OAL, OAM, and OAH (^+^ *p* < 0.001, ^+a^ *p* = 0.004, ^+b^ *p* = 0.001, ^+c^ *p* = 0.003) and the differences between OAG versus OAL, OAM and OAH (*p < 0.001, ^*a^ *p* = 0.001, *^b^ *p* = 0.012) were significant. Results according to one‐way analysis of variance, presented as means with 95% confidence intervals; *n* = 10 rats in each group. (b) The levels of IL‐1β and TNF‐α in serum and IALF. Differences between CG and CH (*p < 0.001, *^a^ *p* = 0.016, *^b^ *p* = 0.005, *^c^ *p* = 0.039), CG and OAG (^+^ *p* < 0.001, ^+a^ *p* = 0.001), and OAG versus OAL, OAM, and OAH (^#^ *p* < 0.001, ^#a^ *p* = 0.028, ^#b^ *p* = 0.012, ^#c^ *p* = 0.003) were significant. Results according to one‐way analysis of variance, presented as means with 95% confidence intervals; *n* = 10 rats in each group. (c) Protein content was determined by western blots of total protein extracted from cartilage. Differences between CG and CH (*p < 0.001, *^a^ *p* = 0.001), CG and OAG (^+^ *p* < 0.001), and OAG versus OAL, OAM, and OAH (^#^ *p* < 0.001, ^#a^ *p* = 0.001, ^#b^ *p* = 0.01) were significant. β‐actin and histone H2A.X were used as internal standards. Results according to one‐way analysis of variance, presented as means with 95% confidence intervals; *n* = 3 rats in each group. Experimental groups: CG; CL, CM, and CH, control group with different durations of treadmill exercise; OAG; and OAL, OAM, and OAH, OA with different durations of treadmill exercise. CG: control group; IALF: intra‐articular lavage fluid; IL‐1β: interleukin‐1β; OAG: OA group; TNF‐α: tumor necrosis factor‐α

There were no significant differences among CG, CL, and CM in the concentrations of TNF‐α and IL‐1β in serum. The serum concentrations of TNF‐α and IL‐1β of CH and OAG were higher than those of CG. However, OAM had decreased serum concentrations of TNF‐α and IL‐1β compared with OAG. The changes in TNF‐α and IL‐1β concentrations in IALF were similar to those observed in serum (Figure 4b).

### 3.3 Western blot analysis

The changes in collagen II, MMP‐13, and NF‐κB p65 in the cartilage in different groups were similar to those observed in histological observations and immunohistochemical analysis (Figure 4c).

**Figure 5:**
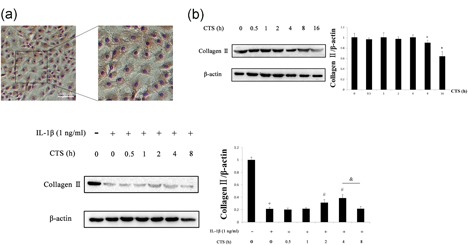
Western blot analysis of chondrocytes. (a) Representative immunohistochemical image of chondrocytes stained with collagen II. Scale bar, 100 μm. Protein content of chondrocytes was determined in western blots according to different durations of CTS (10%, 0.5 Hz) without (b) and with (c) IL‐1β. Differences between normal chondrocytes and chondrocytes subjected to CTS of different durations were significant (*p < 0.001), differences between normal chondrocytes and chondrocytes exposed to IL‐1β were significant (^+^ *p* < 0.001), differences between IL‐1β‐induced chondrocytes and CTS of different durations were significant (^#^ *p* < 0.001), and differences between IL‐1β‐induced chondrocytes with CTS for 4 hr and 8 hr were significant (^&^ *p* < 0.001). β‐actin was used as the internal standard. Results according to one‐way analysis of variance, presented as means with 95% confidence intervals; *n* = 3 rats in each group. Treatment groups: CG; CL, CM, and CH, control group subjected to different durations of treadmill exercise; OAG, OA group; and OAL, OAM, and OAH, OA subjected to different durations of treadmill exercise. CG: control group; CTS: cyclic tensile strain; IL‐1β: interleukin‐1β; OAG: OA group [Color figure can be viewed at wileyonlinelibrary.com]

There was no significant difference in collagen II after 4 hr of CTS (10%, 0.5 Hz), but the content of collagen II in chondrocytes decreased over 8 hr (Figure 5b). However, 4 hr of CTS (10%, 0.5 Hz) ameliorated the changes in collagen II induced by IL‐1β (Figure 5c). Thus, we chose 4 hr of CTS (10%, 0.5 Hz) for further study.

IL‐1β induced decreases in the contents of AMPK‐α1 (phosphor S487) and IκB‐α. CTS (10%, 0.5 Hz, 4 hr) increased the contents of these proteins. Compound C inhibited the increases in levels of these proteins caused by CTS (10%, 0.5 Hz, 4 hr; Figure 7a).

### 3.4 Contents of AMP, ADP, and ATP in chondrocytes

**Figure 6:**
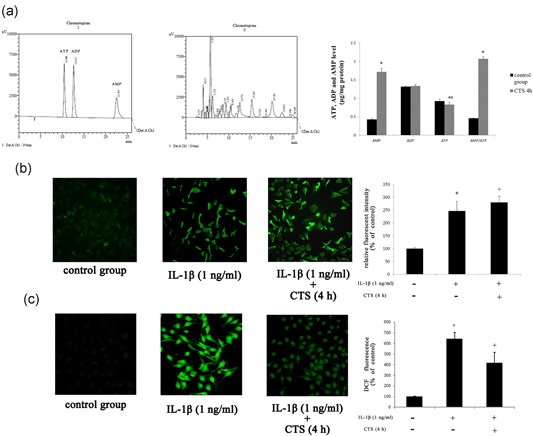
The results of HPLC, Ca^2+^, and ROS analyses in chondrocytes. (a) The contents of AMP, ADP, and ATP of chondrocytes subjected to CTS for 4 hr. Differences between normal chondrocytes and chondrocytes subjected to CTS were significant (*p < 0.001, *p = 0.009). Results according to independent sample *t* test, presented as means with 95% confidence intervals; *n* = 3 rats in each group. The fluorescence microscopy of Ca^2+^ (b) and ROS (c) in chondrocytes. Differences between normal chondrocytes and chondrocytes exposed to IL‐1β were significant (*p < 0.001), and differences between IL‐1β‐induced chondrocytes and those subjected to CTS for 4 hr were significant (^+^ *p* < 0.001). Results according to one‐way analysis of variance, presented as means with 95% confidence intervals; *n* = 3 rats in each group. ADP: adenosine diphosphate; AMP: adenosine monophosphate; ATP: adenosine triphosphate; CTS: cyclic tensile strain; HPLC: high‐performance liquid chromatography; IL‐1β: interleukin‐1β; ROS: reactive oxygen species [Color figure can be viewed at wileyonlinelibrary.com]

The effects of CTS (10%, 0.5 Hz) for 4 hr on AMP, ADP, and ATP contents in chondrocytes were measured by HPLC. We noticed a significant decrease in ATP content, increase in AMP content, and an increase in the AMP/ATP ratio after CTS (Figure 6a).

### 3.5 Intracellular Ca^**2+**^ and ROS analysis of chondrocytes

To evaluate the intracellular Ca^2+^ and oxidative stress of chondrocytes, we measured intracellular Ca^2+^ detected by Fluo‐4AM (Figure 6b) and the production of ROS by DCFH‐DA (Figure 6c).

Intracellular Ca^2+^ and ROS production were enhanced with IL‐1β (1 ng/ml) stimulation. Interestingly, we evaluated the effect of CTS (10%, 0.5 Hz, 4 hr) on ROS generation in chondrocytes exposed to IL‐1β (1 ng/ml) and noticed a significant decrease in ROS level. Intracellular Ca^2+^ was further increased by CTS (10%, 0.5 Hz, 4 hr) stimulation.

### 3.6 Immunofluorescence analysis of chondrocytes

**Figure 7:**
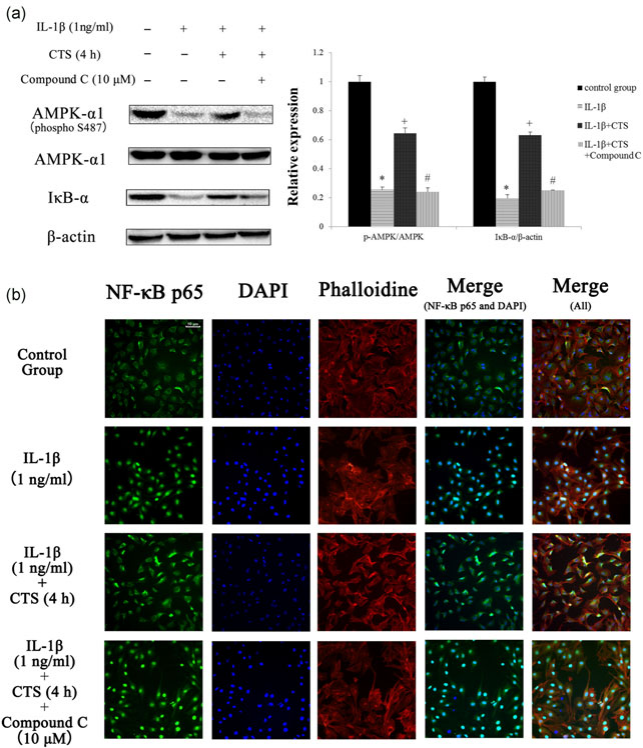
Western blot and immunofluorescence analysis results of NF‐κB p65 in chondrocytes. (a) The results of western blot analysis of AMPK‐α1 (phosphor S487) and IκB‐α. Differences between normal chondrocytes and chondrocytes exposed to IL‐1β were significant (*p < 0.001), differences between IL‐1β‐ induced chondrocytes and those subjected to CTS for 4 hr were significant (^+^ *p* < 0.001), and differences between IL‐1β‐induced chondrocytes subjected to CTS for 4 hr and compound C were significant (^#^ *p* < 0.001). Results according to one‐way analysis of variance, presented as means with 95% confidence intervals; *n* = 3 rats in each group. (b) Effects of CTS for 4 hr on nuclear translocation of NF‐κB p65 in IL‐1β‐induced chondrocytes. The chondrocytes were immunostained using anti‐NF‐κB p65 rabbit antibody (green) and visualized by confocal microcopy. The cytoskeleton was defined by phalloidine (red) and the cell nucleus was defined by DAPI (blue). Scale bar, 50 μm. AMPK: AMP‐activated protein kinase; CTS: cyclic tensile strain; IκB‐α: IκB (inhibitor of NF‐κB)‐α; IL‐1β: interleukin‐1β; NF‐κB: nuclear factor‐κB [Color figure can be viewed at wileyonlinelibrary.com]

A significant nuclear translocation of NF‐κB p65 protein was detected in chondrocytes stimulated with IL‐1β (1 ng/ml) compared with CG (Figure 7b). Compound C (10 μM) reversed the suppression of the nuclear translocation of NF‐κB p65 caused by CTS (10%, 0.5 Hz, 4 hr), which confirmed our hypothesis that CTS (10%, 0.5 Hz, 4 hr) exhibits therapeutic effects by activating AMPK and suppressing nuclear translocation of NF‐κB p65 (Figure 7b).

## 4 DISCUSSION

There were several principal findings of the current study. First, there were no differences in articular cartilage of the knee among CG, CL, and CM groups, but OAM achieved significant therapeutic effects compared to OAG. CH and OAH showed evidence of potential cartilage damage compared with CG and OAG, respectively. Second, CTS had no therapeutic effects on normal chondrocytes and only caused damage under conditions of excessive stress. Moderate CTS (10%, 0.5 Hz, 4 hr) may reduce sensitization to inflammatory response induced by IL‐1β. Third, moderate CTS (10%, 0.5 Hz, 4 hr) could suppress the inflammatory response in chondrocytes induced by IL‐1β via the AMPK/NF‐κB pathway.

To study the connection between mechanical stress and OA progression, we investigated an animal model using rats subjected to treadmill exercise. Intra‐articular injection of MIA induced changes that replicated those observed in humans with OA, including cartilage surface erosion, matrix loss, and inflammation of the synovium (Barve et al., 2007; Cifuentes et al., 2010; Guzman, Evans, Bove, Morenko, & Kilgore, 2003; Schuelert & Mcdougall, 2009). We observed no differences among CG, EL, and EM groups related to the articular cartilage of the knee, including histology (Mankin and OARSI score), protein contents (collagen II, MMP‐13, and NF‐κB p65), or inflammatory mediators (TNF‐α and IL‐1β). Histological changes were ameliorated in OAM, changes in collagen II content in cartilage were reversed, and levels of inflammatory mediators in serum and IALF were reduced. CH and OAH both showed evidence of potential cartilage damage compared with CG and OAG, respectively. Together, our results corroborated findings that OAM could alleviate cartilage damage in knee OA in a rat model (Galois et al., 2004; Na, Kim, Yong, & Hwangbo, 2014; Qian, Liang, Wang, & Wang, 2014).

The mechanisms by which chondrocytes convert biomechanical signals into intracellular events have become an area of intense interest in OA. Thus, we investigated a cellular model of OA by using CTS applied to chondrocytes. The results described intracellular mechanisms by which biomechanical signals are converted into biochemical events. Mechanical signals of CTS (10%, 0.5 Hz) for 4 hr were not perceived as damage to chondrocytes, but CTS (10%, 0.5 Hz) for 8 hr could damage chondrocytes, which was noted as a marked decrease in the content of collagen II. These actions of excessive CTS had damaging effects on chondrocytes similar to those of proinflammatory mediators. These findings further suggest that the duration of CTS was a critical determinant of damage in chondrocytes.

Accumulated evidence suggests that IL‐1β is the pivotal mediator of OA (Bonnelye, Reboul, Duval, Cardelli, & Aubin, 2011). IL‐1β has been associated with the presence of joint inflammation and cartilage destruction. In this study, we used IL‐1β as an inflammatory agent to induce chondrocyte damage: we found that IL‐1β increased in IALF of our OA model. Our findings indicate that different durations of CTS (10%, 0.5 Hz) could induce different results in IL‐1β‐induced chondrocytes. CTS (10%, 0.5 Hz) for 4 hr could alleviate IL‐1β‐induced inflammatory response, but CTS (10%, 0.5 Hz) for 8 hr could aggravate damage in chondrocytes, evidenced by the content of collagen II. Likewise, the present findings are also consistent with observations that treadmill exercise causes histological changes in articular cartilage. It is intriguing that the effects of CTS on chondrocytes are cytokine dependent, because CTS alone failed to achieve therapeutic effects. Our foremost findings are that CTS had no obvious beneficial effects on normal chondrocytes, only causing damage under conditions of excessive stress, but moderate CTS could reduce sensitization to inflammatory response induced by IL‐1β.

**Figure 8:**
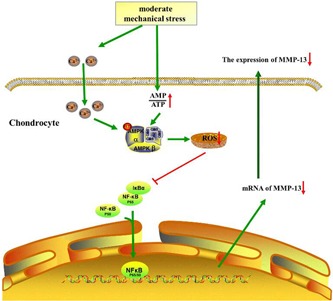
The mechanism of moderate mechanical stress on chondrocytes. Moderate mechanical stress could change the intracellular AMP/ATP ratio and cause Ca^2+^ influx, which activates the AMPK pathway. The activation of AMPK could reduce the level of ROS, which inhibits the nuclear translocation of NF‐κB p65 and, possibly, decrease the content of MMP‐13. AMPK: AMP‐activated protein kinase; AMP: adenosine monophosphate; ATP: adenosine triphosphate; MMP‐13: matrix metalloproteinase‐13; NF‐κB: nuclear factor‐κB; ROS: reactive oxygen species [Color figure can be viewed at wileyonlinelibrary.com]

Another striking finding of the current study is that the AMPK/NF‐κB signal transduction pathway is central to CTS. AMPK serves as a checkpoint to sustain energy balance by modulating biological responses. AMPK is an evolutionarily conserved serine/threonine kinase that was originally identified as the key player in maintaining cellular energy homeostasis (Jeon, 2016). Binding of AMP or ADP causes conformational changes that enhance net phosphorylation at Thr172 and causes allosteric activation (L. Chen et al., 2012). Moderate CTS could activate the AMPK pathway by increasing the intracellular AMP/ATP ratio and causing Ca^2+^ influx, which has been confirmed. Also, the effect of Thr172 in AMPK has been confirmed in many studies (Jeon, 2016). S487 pAMPK antibody also has been used in many studies (Dun, Liu, Zhang, Xie, & Qiu, 2017; Y. Chen et al., 2015). But the phosphorylation at S487 of AMPK in mechanical stress is unknown. And inhibition of AMPK activation significantly impaired mitochondrial function and increased the generation of ROS (Dun et al., 2017; Y. Chen et al., 2015). IL‐1β is a classic proinflammatory cytokine, so it is not surprising that it inhibits the expressions of AMPK‐α1 (phosphor S487) and IκB (inhibitor of NF‐κB)‐α (IκB‐α). In this study, its actions were mediated by NF‐κB p65 nuclear translocation. This is consistent with our observation that CTS (10%, 0.5 Hz) for 4 hr changed the intracellular AMP/ATP ratio and caused Ca^2+^ influx, which activated the AMPK pathway. The activation of AMPK reduced the level of ROS, which inhibited the nuclear translocation of NF‐κB p65. CTS (10%, 0.5 Hz, 4 hr) inhibited nuclear translocation of NF‐κB p65 via the AMPK signal pathway, which was confirmed by analysis of compound C. Hence, the anti‐inflammatory actions of CTS (10%, 0.5 Hz) for 4 hr were mediated both by activating the AMPK signal pathway and by inhibiting NF‐κB nuclear translocation in IL‐1β induced chondrocytes. The activation of the AMPK/NF‐κB pathway nullifies the increased MMP‐13 production induced by IL‐1β, which inhibits collagen II breakdown in chondrocytes (Figure 8). Interestingly, the AMPK/NF‐κB pathway may explain the reason why moderate CTS had different effects on chondrocytes between models with and without IL‐1β. Because NF‐κB p65 exists in the cytoplasm under normal conditions, moderate CTS did not achieve therapeutic effects via the AMPK/NF‐κB signaling pathway.

This study has several limitations that must be considered. First, further study is needed to explore different conditions (such as intensity and frequency) that cause damage to articular cartilage and chondrocytes. Second, CTS is two‐dimensional loading: chondrocytes are strained in a monolayer and only one surface is elongated. We will continue to investigate these issues in future studies.

In summary, we used treadmill exercise in rats as an animal model and CTS applied to chondrocytes as a cellular model to explore the effects of mechanical stress in OA. Our findings indicate that mechanical stress had no therapeutic effects on normal articular cartilage and chondrocytes: mechanical stress only caused damage under excessive stimulation. However, moderate mechanical stress could reduce sensitization to inflammatory responses of articular cartilage and chondrocytes through the AMPK/NF‐κB pathway. Our results not only provide crucial leads to unveiling the effects of mechanical stress on articular cartilage and chondrocytes but also provide molecular evidence for biochemical signals generated by mechanicalstress.

## CONFLICTS OF INTEREST

All authors declare that there have no competing interests to this manuscript.

## AUTHOR CONTRIBUTIONS

Y. Yang and L. Bai contributed to the conception and design. Y. Yang, Y. Wang, X Zhang and Y. Gang involved in the treadmill exercise experiment. Y. Yang, Y. Wang and H. Zhang involved in cell cultures and cyclic tensile strain. All authors contributed to the acquisition and analysis of data. Y. Yang, Y. Wang and Y. Kong involved in statistical analysis and manuscript preparation. L. Bai conceived the final approval of the version to be submitted and obtaining of funding. All authors contributed to revising the manuscript critically for important intellectual content, and approved the manuscript for publication.
